# Supplementary material for: Altered microRNA Transcriptome in Cultured Human Liver Cells upon Infection with Ebola Virus
Source: Int J Mol Sci. 2021 Apr 6;22(7):3792. doi: 10.3390/ijms22073792 (PMC8038836; doi:10.3390/ijms22073792)
Supplement: Supplementary file 1 [file ijms-22-03792-s001.zip › Supplementary_File/C_ GO_Analysis_Results/16-30nt_go_Reston-96h-Huh7_vs_Control-96h-Huh7_up.mature_mirna_targets/BP_Count.pdf]

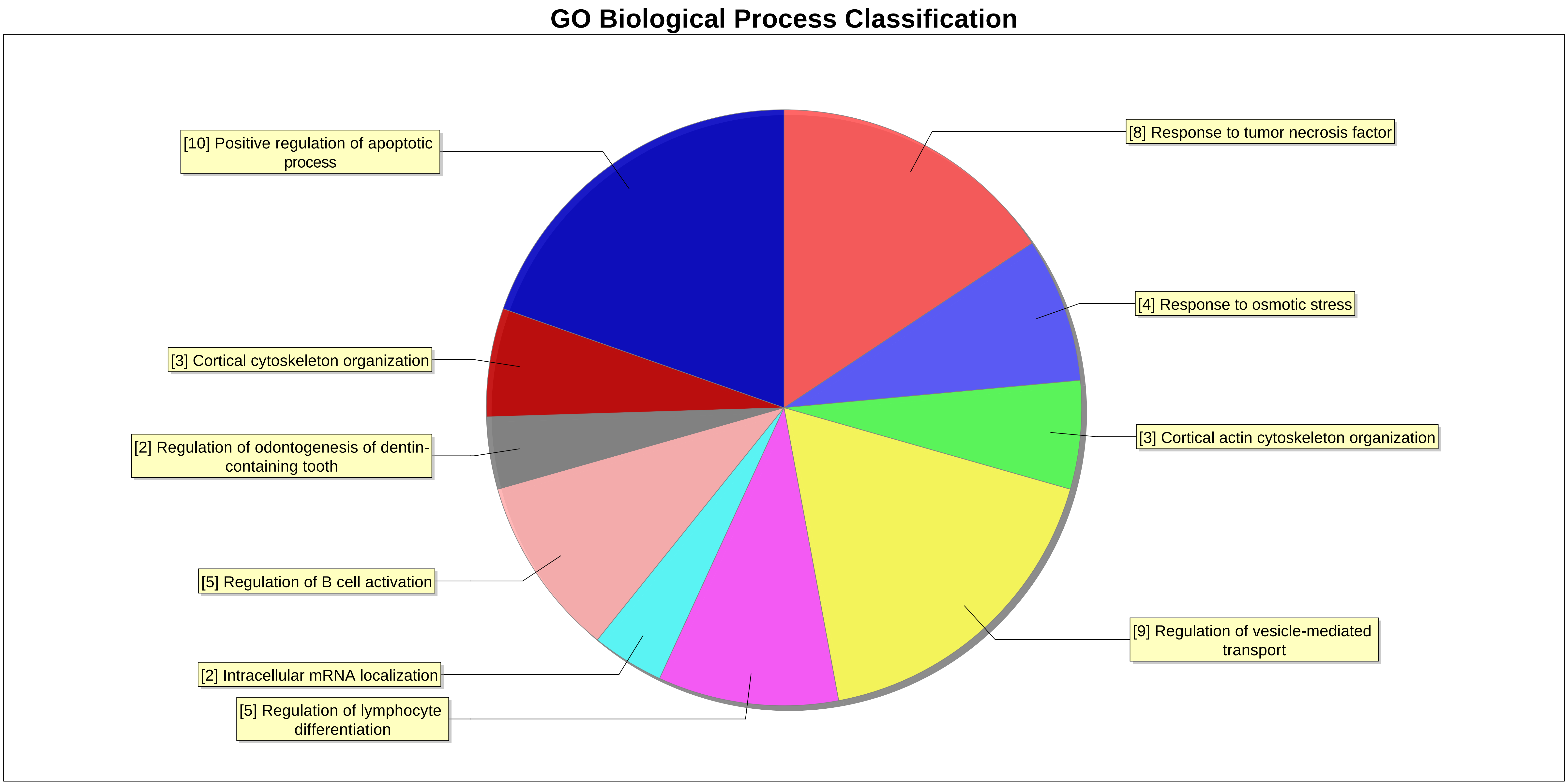
GO Biological Process Classification
[10] Positive regulation of apoptotic process
[8] Response to tumor necrosis factor
[4] Response to osmotic stress
[3] Cortical cytoskeleton organization
[3] Cortical actin cytoskeleton organization
[2] Regulation of odontogenesis of dentin-containing tooth
[5] Regulation of B cell activation
[9] Regulation of vesicle-mediated transport
[2] Intracellular mRNA localization
[5] Regulation of lymphocyte differentiation
